# Eczema Genitale

**Published:** 1873-04-01

**Authors:** James N. Hyde

**Affiliations:** Chicago


### ECZEMA GENITALE.

BY JAMES N. HYDE, M.D., CHICAGO.

An exceedingly common and distressing cutaneous affection is that called eczema genitale, or eczema pudendi. It is described by Erasmus Wilson and Tilbury Fox as characterized by the phenomena peculiar to catarrhal or eczematous inflammation of the integument in all portions of the body. It ordinarily is first developed upon the scrotum, and extends to contiguous patches of the thighs, anus, or perineum ; or, commencing in the latter situation, it spreads subsequently to the scrotum. It is accompanied by intolerable pruritus, increased rather than diminished by the efforts of scratching. The vesicles usually burst or are ruptured as soon as formed ; copious ichorous secretion is effused ; fissures and excavations result. It assumes the chronic form, lasting for months and years, and is peculiar to the middle period of life. In females it is, if possible, more distressing than in males, and is complicated by mucous surfaces becoming affected ; the vulval irritation being aggravated by morbid vaginal secretions, which come in contact with its lining membrane. The functions of the region are deranged, the smarting and pruritus becoming excessive. Children are rarely affected. Fox adds, that the scrotum is often thickened, puckered, moist and tender. Sometimes it becomes covered with large, thin scales, the fluid oozing freely from numerous fissures between them. The affection is termed eczema nummularis, and lepra vulgaris, when the patches occur in the shape and of the size of coins of metal.

There is a sub-variety of this local disease, which is so well marked in its causation and phenomena that one is tempted to give it a special name, even though he have in view the charge that "dermatology consists of a nomenclature."

It occurs almost exclusively in middle life, and is characterized by a catarrhal affection of the surfaces bordering upon the anus, with a predilection for the integument in the cleft of the nates. It is essentially chronic in its course ; lasts for months and even years ; and *exhibits no tendencg to spread or extend* to neighboring surfaces. The occurrence of vesiculation, as Wilson intimates, is rarely observed at the time of clinical examination, such stages having been succeeded by papular or scaly eruption. There is reason to believe that the sebiferous system is usually implicated.

The history of patients thus affected is frequently this : They noticed, at first, a slight irritation or itching of the parts in the neighborhood of the anus. For relief they have made trial of various external medicaments, obtained of druggists or suggested by friends. These proved of no avail ; the eruption became chronic; the implicated surface did not increase in extent ; but the itching became so severe and intolerable, especially after becoming warmed in bed at night, that this induced them to seek professional assistance. And often they have rung the changes on lotions, ointments, powders and internal preparations, till they despair of relief.

Patients of this class are not such as are generally catalogued subjects of eczema. The disease occurs equally among those who do and those who do not pay scrupulous attention to personal cleanliness ; in the emaciated and the obese ; in those who are otherwise unconscious of physical ailment, as well as in those who suffer from dyspepsia or constitutional contamination. It is also found in persons of all temperaments.

At present, dermatologists are agreed that eczematous or catarrhal forms of cutaneous disease depend upon faulty innervation. And it is not necessary to believe that this is always a cause which obtains generally ; "faulty innervation" may be true only of the area supplied by a single nerve-twig. Podcopaew has demonstrated the existence of fine plexuses of nerves external to the nervous papillæ, and forming a network in the rete-mucosum of the epidermis. It can then be seen how readily external irritants may produce a nerve-paresis, and how the metamorphosis of cells in the cuticle is more dependent upon vital than mechanical forces.

They who have noticed the fetor of pus in abscesses located in the recto-ischial space, and particularly in abscesses where no communication was established with the gut, can readily believe that mere proximity of stercoraceous substance is sufficient to excite irritation in neighboring parts. The use of printed newspaper after stool is probably another cause of this form of eczema. Such a habit has been assigned in order to account for the production of external hæmorrhoids also ; but this can be shown to be an error. When the tissues adjoining the anus are engorged with blood, as a result of effort in defecation, one can hardly overestimate the injurious effects of contact with a sheet of paper which has been passed over rollers lubricated with kerosene, and impressed with an ink manufactured of lamp-black and linseed oil (see for example the *Evening Journal* of our city). Bathing in warm or hot water certainly aggravates, and perhaps predisposes to this complaint.

Indications for treatment are found in considering the pathology of this affection ; and the results of a properly directed medication in cases which have lasted for years is as surprising as it is satisfactory. Soothing applications are requisite, of such a character that they protect the parts from contact with external irritants, and *influence directly the nerves* in the vicinity, while exercising an alterative action upon the skin and such of its accessories as are concerned in the disorder. Now, such an external application should be made in the form of an ointment, inasmuch as the active factors of irritation are almost constantly present; and ointments can be applied in such a way as that the diseased skin can be completely protected from the time of one application to another; while the application of lotions and powders in this region has an effect which is more or less transitory, according as the patient is or is not engaged in his ordinary occupations.

I have succeeded in giving complete relief to the last six patients who have consulted me with reference to this complaint, after a few applications of the ointment of belladonna, combined with the sulpho-carbolate of zinc. Such a preparation seems to meet fully the indications given. The use of the zinc-salt was suggested to me last year by a friend, a physician, who had succeeded in relieving himself, after making trial of various preparations for years, of an eczematous patch upon the scrotum.

I am, however, inclined to believe that the belladonna is the more useful agent in this combination ; not only because of its relief of the intolerable itching, which is the result of the nervous derangement in the site of the disease, but also because I have seen painful syphilitic condylomata disappear under its use (as well as eczema genitale) when the drug was given without combination.

It would hardly seem necessary to call the attention of the profession to these details, were it not for the fact that belladonna is *not* very generally recommended in cutaneous affections. Of one hundred and ninety excellent formulæ appended to the very practical treatise of Tilbury Fox, on "Skin Diseases," one only contains belladonna as a component, and that recommends its use in solution with glycerine and hydrocyanic acid, "for papular and phlegmonous affections." In the body of this work the remedy is alluded to once in connection with the treatment of catarrhal inflammation of the skin. Dr. McCall Anderson, though he discusses exhaustively and with great ability all medications advised in the treatment of this disease, is equally silent on the subject of belladonna.

If any one will take the trouble to examine the U. S. Dispensatory on this subject, he will discover that the enumeration of the "medical properties and uses" of this medicament as an external application, includes merely its availability in schirrhous tnmors, and cancerous or ill-conditioned ulcers ; in certain disorders of the mammæ, uterus, bowels, urethra, bladder and sphincters ; and especially in cases where there is tension, fissure, spasm or undue tonicity of fibre. No reference is made to its therapeutic value in cutaneous disorders. Nor do the French authorities give details on this point. Hirtz, in a monograph before me, on the subject of belladonna, makes no allusion to such applications in skin diseases, though careful to enumerate every other possible use of this valuable agent, in the fulfillment of two leading indications—relief of pain and resolution of spasm.

It is possible that the appearance of its *medicinal rash*, after an internal exhibition, may have tended to produce a belief that the remedy is counter-indicated, when an eruption already exists ; but such an opinion can be readily shown to be intenable.

In prescribing the preparations of belladonna, it is advisable to order an extemporaneous ointment, rather than the officinal, which contains sixty grains of the extract to the ounce of lard. Apart from the consideration that the former will be very much more likely to be newly compounded than the latter, it will be advantageous to change the quantity of extract in each ounce, according to the severity of the itching, and the grade of the disease. A scruple of the extract to the ounce of lard and cerate, will occasionally prove sufficient to allay the irritation in moderate cases. The sulpho-carbolate of zinc, as prepared by Messrs. Powers & Weightman, of Philadelphia, is now kept in most of our drug stores, and may be used in doses similar to those of the ordinary sulphate, from half a grain to ten grains (and even more) being incorporated into ointments, according to the locality and disease to which they are to be applied.

